# Pan HDACi Valproic Acid and Trichostatin A Show Apparently Contrasting Inflammatory Responses in Cultured J774A.1 Macrophages

**DOI:** 10.3390/epigenomes6040038

**Published:** 2022-11-03

**Authors:** Ubah Dominic Babah Ubah, Korawin Triyasakorn, Brandon Roan, Minsyusheen Conlin, James C. K. Lai, Prabha S. Awale

**Affiliations:** 1 Department of Biomedical and Pharmaceutical Sciences, College of Pharmacy, Idaho State University, 921 S 8th Avenue, Mail Stop 8288, Pocatello, ID 83209, USA; 2 Division of Health Sciences, Idaho State University, 921 S 8th Avenue, Mail Stop 8288, Pocatello, ID 83209, USA; 3 Department of Biological Sciences, Idaho State University, 921 S 8th Avenue, Mail Stop 8288, Pocatello, ID 83209, USA

**Keywords:** valproic acid, trichostatin-A, IL-1β, TNF-alpha, NO, J774A.1, macrophages, histone deacetylase inhibitors

## Abstract

This study was initiated as an attempt to clarify some of the apparent conflicting data regarding the so-called anti-inflammatory versus proinflammatory properties of histone deacetylase inhibitors (HDACis). In cell culture, typically, chronic pretreatment with the HDACi valproic acid (VPA) and trichostatin A (TSA) exhibits an anti-inflammatory effect. However, the effect of acute treatment with VPA and TSA on the levels of inflammatory cytokines in J774A.1 macrophage cell line is unknown. Therefore, this study investigated the effect of acute treatment with VPA and TSA on levels of key inflammatory cytokines in maximally stimulated J774A.1 cells. J774A.1 macrophages were treated with either VPA or TSA for 1 h (acute treatment), followed by maximal stimulation with LPS + IFNγ for 24 h. ELISA was used to measure the levels of proinflammatory cytokines TNFα, NO and IL-1β from the culture medium. Acute treatment with VPA showed a dose-dependent increase in levels of all three cytokines. Similar to VPA, TSA also showed a dose-dependent increase in levels of IL-1β alone. This study sheds new light on the conflicting data in the literature that may partly be explained by acute or short-term exposure versus chronic or long-term exposure to HDACi.

## 1. Introduction

The many apparently conflicting data in the literature regarding the so-called anti-inflammatory versus proinflammatory properties of histone deacetylase inhibitors (HDACi) may be attributed to the fact that the molecular mechanisms underlying such inhibitors’ pro- and anti-inflammatory effects are poorly understood and incompletely elucidated. This study was initiated to address some of this critical gap in the literature by employing J774A.1 macrophages, which constitute a characterized immune cell line in vitro.

HDACis are increasingly being investigated for their potential as anti-inflammatory agents because increasing evidence suggests that an imbalance in histone acetylases (HATs) and histone deacetylases (HDACs) could be pathophysiologically/pathogenetically implicated in diseases like cancer and inflammation [1]. For example, increased HATs but decreased HDAC activities in peripheral blood monocytes are associated with neutrophilic airway inflammation [2]. Consistent with this observation, decreased activities and mRNA expression, specifically of HDAC1 and 2 in monocytes, were noted in asthmatic patients [3]. This putative involvement of HATs and HDACs in these inflammatory diseases notwithstanding, the roles of these enzymes in other chronic inflammatory diseases have not been comprehensively or systematically assessed. Nevertheless, recent in vivo studies in several animal models of inflammation (including those of autoimmune diseases and traumatic injury) suggest HDACi could be anti-inflammatory agents: for example, the pan HDACi valproic acid (VPA), at 500 mg/kg, induced decreases in mRNA levels of interleukin one beta (IL-1β), tumor necrosis factor-alpha (TNFα), inducible nitric oxide synthase (iNOS), measured on day 13 and day 15, respectively, of treatment in both the preventative and therapeutic models of autoimmune encephalomyelitis (EAE) [4]. Similarly, in a traumatic spinal cord injury (SCI) model, three days of treatment with VPA (300 mg/kg/Day) induced a significant reduction in TNFα, IL-1β, IL-6 and IFNγ [5]. This anti-inflammatory mechanism was attributed to the VPA-induced decrease of the histone protein HDAC3, which was reportedly induced by SCI. Furthermore, in the same model, VPA also increased the acetylation of nonhistone proteins nuclear factor kappa Bp65 (NF-kBp65) and signal transducer and activator of transcription (STAT1), thereby lowering the transcriptional activities of the STAT1 and NF-kB complex and reducing the microglia-mediated inflammation in this model [5]. 

Consistent with its effects in vivo mentioned above, similar anti-inflammatory effects of VPA have been observed in both immortalized and primary macrophage cell cultures. Pretreatment of RAW264.7 cells and primary bone marrow-derived macrophages (BMDM) from C57BL/6 and BALB/C mice with doses ranging from 0.5 mM to 2 mM of VPA followed by subsequent stimulation with LPS alone caused a dose-dependent reduction in TNFα at both the transcript and protein levels in both the cell line and the primary culture [6]. Chen et al. noted a similar dose-dependent reduction in TNFα and nitrite levels when a rat mesencephalic neuron glial culture was pretreated with 0.05 mM–0.6 mM VPA, with subsequent LPS stimulation [7]. Furthermore, several other HDACis, including the pan HDACis sodium butyrate, SAHA, TSA and isoenzyme selective HDACis such as MS-275, KBH-A42, RGFP966, reportedly induce anti-inflammatory effects in both in vitro and in vivo inflammatory models [8,9,10,11,12,13,14]. Thus, these findings suggest these compounds may serve as promising new candidates for the treatment of inflammation.

**Figure 1:**
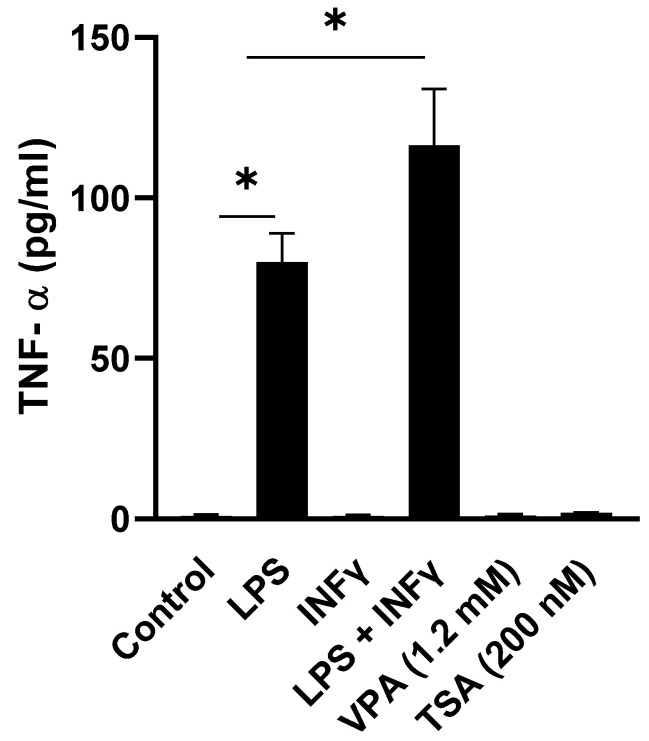
HDACis VPA and TSA by themselves have no effect on the release of TNFα from macrophages. J774A.1 cells were stimulated with LPS (1 µg/mL) alone, IFNγ (1 ng/mL) alone, LPS (1 µg/mL) + IFNγ (1 ng/mL), VPA (1.2 mM) and TSA (200 nM). TNFα levels in culture media were measured at 24 h post-exposure and were expressed as pg/mL. Data are presented as mean ± SD from 3 independent experiments (10 replicates each). * *p* ˂ 0.05 by Student’s *t*-test vs. control.

Despite the generally accepted anti-inflammatory effects of HDACis as alluded to above, there is evidence that HDACis also exert some pro-inflammatory effects: interestingly, in almost all cases, their anti-inflammatory effects in vitro have been attributed to chronic or prolonged pre-exposure of immune cells to the HDACi ranging anywhere between 4 and 48 h with subsequent stimulation with a commonly used inflammation-inducing agent, namely, lipopolysaccharide (LPS). On the other hand, very few studies report on the acute pro-inflammatory effects of HDACis: these are sporadic and limited by employing only one or two doses of these agents. More importantly, the experimental paradigm of employing a single inflammation-inducing agent like LPS has definite limitations in that the outcome does not accurately or realistically represent what happens in vivo for the following reasons. LPS treatment in vivo is a potent inducer of the critical transcription factor NF-ĸB, leading to the secretion and release of inflammatory mediators and cytokines [15,16]. These initial inflammatory cytokines, in turn, stimulate their cognate receptors in an autocrine manner, thereby amplifying the inflammatory response. Thus, to overcome the limitations of the paradigm of employing a single inflammation-inducing agent and to closely mimic the in vivo LPS-induced pathophysiological sequelae in cell culture studies in vitro, a new paradigm can include treatment of cells with LPS combined with IFNγ, which primes the cells to amplify their response to LPS through increasing the number of toll-like receptor 4 (TLR4) receptors, thereby ultimately giving rise to a “maximal stimulation” of inflammation [17,18]. Indeed, consistent with the pathophysiological relevance of this new “maximal stimulation” paradigm, our preliminary results (see Figure 1) prompted us to employ the combination of LPS + IFNγ to stimulate cultured macrophages because the combination induced more than an additive effect on eliciting the response in the levels of proinflammatory cytokines TNFα, IL-1β and NO compared to LPS alone (see Results). Furthermore, this study employing the new paradigm addresses another critical gap in the literature because the effect of acute treatment of VPA on cytokine levels in maximally stimulated macrophages is unknown.

Brain development is complex process involving multiple cell types, hormones and signaling molecules like cytokines, although the physiological roles of the various cells, hormones and signaling molecules underlying this complex developmental process are still poorly understood. Exposure to VPA (an HDACi and immunomodulatory agent) during pregnancy is associated with a high risk of neurodevelopmental disorder autism [19]. We have previously shown that embryonic exposure to a single dose of VPA (acute treatment) induces a significant decreases in the number of the resident macrophages (namely microglia) in the primary motor cortex of the brain during early neonatal development in a murine BALB/c VPA model of autism, although the underlying mechanism of these VPA-induced decreases in microglial cell number has not been fully elucidated [20]. Nonetheless, an emerging mechanistic possibility that the reduction of microglial number may be a secondary effect of the drug (preliminary studies and unpublished data) likely attributable to increased inflammatory cytokines and mediators induced by VPA has provided another mechanistic rationale for this study. We initiated this study to further investigate the putative effects of acute treatment of VPA using an extensive range of doses on cytokine levels in maximally stimulated macrophages in culture with the notion that the outcome of this study may improve our understanding of the putative mechanism underlying the VPA-induced reduced microglial cell number in the developing brain, ultimately shedding some new light on the apparent contrasting nature of the anti- and pro-inflammatory effects of VPA. 

This study aimed to investigate our hypothesis that acute treatment of cultured J774A.1 macrophage cells with an extensive range of doses of VPA followed by our novel “maximal stimulation” induces VPA dose-related decreases in the levels of cytokines in the culture medium. Additionally, to further investigate this hypothesis and hopefully provide additional clarification of the apparent anti- and pro-inflammatory effects of HDACi, we also included parallel experiments employing the same experimental paradigm but with an extensive range of doses of another chemically distinct pan HDACi trichostatin A (TSA) for comparison.

## 2. Results

Our recent study demonstrated that embryonic exposure to a single dose (acute exposure) of VPA induced a significant decrease in the number of resident macrophages (namely microglia) in the central nervous system (CNS) during early neonatal development in our murine BALB/c VPA model of autism although the mechanisms responsible for inducing the decreases in CNS microglial numbers in our murine VPA model of autism remain to be elucidated [20]. This exciting finding prompted us to employ the J774A.1 macrophage cell line derived from the BALB/CN mouse (which shows some genetic origin and phenotypic characteristics similar to those of the BALB/c mouse employed to generate our VPA model of autism) in this study to investigate our hypothesis that acute treatment of cultured J774A.1 macrophage cells with an extensive range of doses of VPA followed by our novel “maximal stimulation” induces VPA dose-related decreases in the levels of cytokines. This study focused on elucidating the effects of the two HDACis on the levels of proinflammatory cytokines TNFα, IL-1β and NO by cultured J774A.1 macrophage cells. For our treatment paradigm, we primed J774A.1 macrophages with either VPA or TSA for one hour and therefore, from now on, we will refer to this priming as “acute treatment” subsequently followed by maximal stimulation with LPS + IFNγ and henceforth we will refer to this as “maximal stimulation” in this paper.

We began this study by determining whether or not treatment of cultured J774A.1 macrophage cells with the HDACis VPA or TSA alone might influence the levels of proinflammatory cytokines. Our results indicated that treatment of cultured J774A.1 macrophage cells with the highest dose of VPA (1.2 mM) or TSA (200 nM) alone for 24 h did not significantly influence the levels of TNFα (Figure 1), NO and IL-1β (data not shown) in culture medium. By contrast and as expected, exposure of J774A.1 macrophage cells to LPS alone induced significant increases in the levels of TNFα (to 80 ± 9 pg/mL) (Figure 1) in the culture medium. While treatment of the cultured J774A.1 macrophage cells with the proinflammatory mediator INFγ by itself had a negligible effect on the levels of TNFα (Figure 1), NO and IL-1β (data not shown), the combination treatment of these cells with LPS + IFNγ induced apparent additive effects on the levels of TNFα (to 116 ± 17 pg/mL) (Figure 1), NO and IL-1β (data not shown). Thus, these results strongly suggested the apparent additive effect achieved by employing this combination treatment (LPS + IFNγ) provided us with a more pathophysiological relevant rationale for employing this “maximal stimulation” paradigm for subsequent experiments to elucidate the putative pro- and/or anti-inflammatory effects of the HDACi in the cultured macrophage cells. 

### 2.1. TNFα Levels Are Differentially Altered by Acute Treatment with HDACis VPA and TSA in Maximally Stimulated J774A.1 Cells

**Figure 2:**
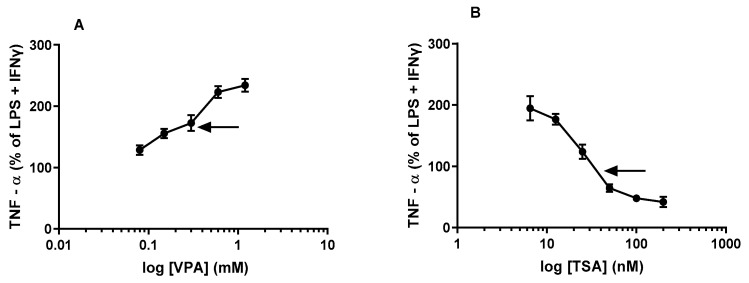
HDACis VPA and TSA alter TNFα release from stimulated macrophages at 6 h: J774A.1 cells were treated with either VPA (**A**) at concentrations ranging from 0.075 mM to 1.2 mM or TSA (**B**) at concentrations ranging from 6.5 nM to 200 nM for one hour, prior to LPS (1 µg/mL) + IFNγ (1 ng/mL) stimulation for 6 h. The data from LPS + IFNγ stimulated J774A.1 cells are expressed as a percentage of the corresponding values for levels of TNFα (set as 100%) obtained. The apparent ED_50_ value for VPA is ~0.3 mM, indicated by the arrow—see Figure 2; the apparent IC_50_ value for TSA is ~40 nM, indicated by the arrow—see Figure 2B. See M&M for the calculation of ED_50_ and IC_50_ values. Data are presented as mean ± SD from 3 independent experiments (10 replicates each). All doses of VPA & TSA used in this study reached significance (*p* ˂ 0.05, by ANOVA and post hoc Student Newman–Keuls test) from the corresponding value in control (LPS + IFNγ).

As mentioned in the Introduction, even though the HDACis VPA and TSA are known to be immunomodulatory agents, the very few studies investigating their acute pro-inflammatory effects reportedly only employed one or two HDACi doses and a single inflammation-inducing agent, namely LPS. This study was designed to overcome the limitations of the latter studies and to address the critical gaps in the literature by elucidating the effects of acute exposure of cultured J774A.1 macrophage cells to a range of concentrations of VPA and TSA on their production and release of cytokines subsequent to our maximal stimulation with the combination treatment employing LPS + IFNγ. Current literature suggests that TNFα can be detected in the culture medium as early as 1 h after LPS stimulation but it peaks between 4 and 8 h, after which it begins to decline but still remains high (at 24 h) relative to the levels of TNFα detected after 2 h of LPS stimulation [21]. Based on the time course, we chose two time points in this study. The 6 h time point was selected to measure peak levels of TNFα and represents a short-term effect. The 24 h time point (long-term effect) was chosen to determine if the putative dose-related effects of the acute treatment with the HDACis VPA and TSA on TNFα levels released into the culture medium might differ from the corresponding effects obtained at 6 h (see Figure 2) after maximal stimulation.

**Figure 3:**
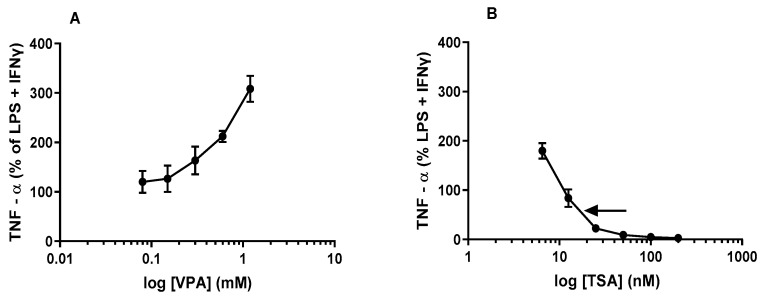
HDACis VPA and TSA alter TNFα release from stimulated macrophages at 24 h: J774A.1 cells were treated with either VPA (**A**) at concentrations ranging from 0.075 mM to 1.2 mM or TSA (**B**) at concentrations ranging from 6.5 nM to 200 nM for one hour, prior to LPS (1 µg/mL) + IFNγ (1 ng/mL) stimulation for 24 h. The data from LPS + IFNγ stimulated J774A.1 cells are expressed as a percentage of the corresponding value for the levels of TNFα (set as 100%) obtained. The apparent IC_50_ value for TSA is ~18 nM, indicated by the arrow; see Figure 3B. See M&M for the calculation of IC_50_ values. Data are presented as mean ± SD from 3 independent experiments (10 replicates each). Except for 0.08 mM VPA, all doses of VPA and TSA used in this study reached significance (*p* ˂ 0.05, by ANOVA and post hoc Student Newman–Keuls test) from the corresponding value in control (LPS + IFNγ).

Indeed, consistent with our initial notion, the kinetic concentration-response curve confirms there may be time-dependent changes in the concentration response to the levels of TNFα. The results shown in Figure 2A clearly demonstrated that acute treatment with various concentrations of the HDACi VPA induced an apparent significant (*p* < 0.05; Figure 2) concentration-related linear stimulation of the TNFα into the culture medium by the J774A.1 cells at 6 h subsequent to maximal stimulation. The apparent ED_50_ for VPA in inducing the stimulation of the TNFα levels at 6 h was ~0.3 mM (Figure 2A, see arrow). On the other hand, the concentration-response curve for the longer (24 h) exposure time is significantly different from the shorter (6 h) exposure time. While the long-term (24 h) effect on TNFα levels is still apparently stimulatory, the kinetics appears to be nonlinear and at the therapeutic range of VPA employed in this study, the highest concentration of VPA apparently did not reach saturation and therefore we did not derive the ED_50_ (Figure 3A).

In contrast to the consistent concentration-related stimulatory effects of VPA, acute treatment with different concentrations of TSA followed by maximal stimulation exhibited a markedly different response from VPA. Both the short-term (6 h) and long-term (24 h) effects of acute treatment with TSA followed by maximal stimulation induced somewhat similar biphasic responses (See Figure 2B and Figure 3B), but with some very significant differences. The results showed that the short-term (6 h) effect of acute treatment of J774A.1 cells with different concentrations of TSA followed by maximal stimulation induced significant stimulation of TNFα at lower concentrations (6.5 nM–25 nM, see Figure 2B) but showed a concentration-related inhibitory effect (with an apparent IC_50_ value of ~40 nM, indicated by the arrow, see Figure 2B) on the TNFα levels with increasing concentrations (30 nM–200 nM) of TSA. At the highest concentration of TSA (200 nM, see Figure 2B) used in this study, TSA apparently did not completely abolish the release of TNFα from J774A.1 cells following maximal stimulation.

Consistent with the short-term effect, our results (see Figure 3B) indicate that the long-term (24 h) effect of acute treatment of J774A.1 cells with low concentrations (6.5 nM–10 nM) of TSA with subsequent maximal stimulation appears to be stimulatory. However, at TSA concentrations of 10 nM and higher, our results clearly demonstrated significant concentration-dependent inhibitory effects on TNFα levels (with an apparent IC_50_ ~18 nM, see Figure 3B) in the culture medium from J774A.1 cells. In contrast to the short-term (6 h, see Figure 2B) effect, where the highest concentration of TSA apparently did not completely abolish the release of TNFα from J774A.1 cells following maximal stimulation, the long-term exposure time combined with a high concentration (100 nM) of TSA had abolished entirely the production and release of TNFα (Figure 3B) in culture medium from J774A.1 cells.

### 2.2. NO Levels Are Differentially Altered by Acute Treatment with HDACis VPA and TSA in Maximally Stimulated J774A.1 Cells

A downstream component of macrophage activation is the induction of iNOS. Nitric oxide (NO) is an important inflammatory component of iNOS in activated macrophages [22,23]. Inflammatory stimuli like IFNγ, TNFα IL-1β and bacterial LPS are known to cause an increase in iNOS induction, resulting in large quantities of NO production [24,25,26]. Data in the literature suggest that upon stimulation of macrophages with LPS + IFNγ, macrophages exhibited a lag time (4–12 h) in the synthesis of nitrite/nitrate but subsequently proceeded at a linear rate for 36 to 42 h [27]. These time course studies prompted us to evaluate the effects of acute treatment of the HDACis VPA and TSA with subsequent maximal stimulation on NO levels in J774A.1 cells at 24 h that corresponded with sustained linear rates of NO levels from maximally stimulated macrophages. 

**Figure 4:**
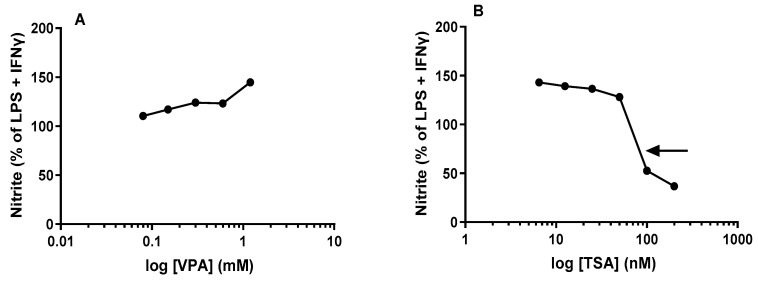
HDACis VPA and TSA have a modest effect on NO release from stimulated macrophages at 24 h: J774A.1 cells were treated with either VPA (**A**) at concentrations ranging from 0.075 mM to 1.2 mM or TSA (**B**) at concentrations ranging from 6.5 nM to 200 nM for one hour, prior to LPS (1 µg/mL) + IFNγ (1 ng/mL) stimulation for 24 h. The data from LPS + IFNγ stimulated J774A.1 cells are expressed as a percentage of the corresponding value for the levels of NO (set as 100%) obtained. The apparent IC_50_ value for TSA is ~80 nM, as indicated by the arrow, see Figure 4B. See M&M for the calculation of IC_50_ values. Data are presented as mean ± SD from 3 independent experiments (10 replicates each). Except for 0.08 mM and 0.15 mM VPA, all doses of VPA and TSA used in this study reached significance (*p* ˂ 0.05, by ANOVA and post hoc Student Newman–Keuls test) from the corresponding value in controls (LPS + IFNγ).

The results shown in Figure 4A demonstrate that acute treatment with different concentrations of VPA induced a modest but significant increase in NO levels in culture medium from maximally stimulated J774A.1 cells at 24 h. At all doses ranging from 0.075 mM to 1.2 mM, VPA induced an overall concentration-dependent stimulation of NO in the culture medium by J774A.1 cells and at the therapeutic range of VPA employed in this study, the highest concentration of VPA apparently did not reach saturation and therefore we did not derive the ED_50_ (Figure 4A). At the highest concentration of TSA (200 nM, see Figure 2B) used in this study, TSA apparently did not completely abolish the release of TNFα from J774A.1 cells following maximal stimulation.

In contrast to VPA, which induced a general stimulatory effect on NO levels in J774A.1 cells, acute treatment with different concentrations of TSA with subsequent maximal stimulation exhibited a very interesting biphasic response. At lower concentrations (6.5 nM–50 nM), TSA induced and maintained a sustained but almost constant stimulatory effect (Figure 4B). However, at higher concentrations beginning at 50 nM, TSA started to induce an apparent inhibitory effect (with an apparent IC_50_ ~80 nM indicated by the arrow, see Figure 4B). Moreover, at the highest concentration of TSA (200 nM, see Figure 4B) used in this study, TSA apparently did not completely abolish the release of NO from J774A.1 cells following maximal stimulation but brought it down to about 30% inhibition.

### 2.3. IL-1β Is Significantly Potentiated by Acute Treatment with HDACis VPA and TSA in Maximally Stimulated J774A.1 Cells

Another key inflammatory mediator largely overlooked in the inflammatory response of HDACi is IL-1β. IL-1β is also released slowly over 24–48 h along with TNFα and NO and mediates fever and hyperalgesia upon stimulation of macrophages/monocytes in vivo [28]. Therefore, we wanted to investigate the effect of acute pretreatment of the HDACis VPA and TSA on the production of IL-1β levels from maximally stimulated macrophages.

**Figure 5:**
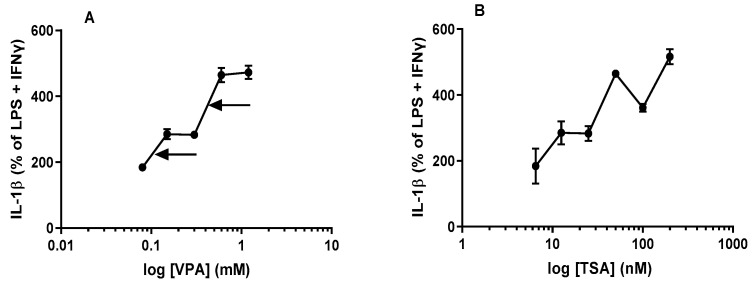
HDACis VPA and TSA significantly potentiate IL-1β release from stimulated macrophages at 24 h: J774A.1 cells were treated with either VPA (**A**) at concentrations ranging from 0.075 mM to 1.2 mM or TSA (**B**) at concentrations ranging from 6.5 nM to 200 nM for one hour, prior to LPS (1 µg/mL) + IFNγ (1 ng/mL) stimulation for 24 h. The data from LPS + IFNγ stimulated J774A.1 cells are expressed as a percentage of the corresponding values for the levels of IL-1β (set as 100%) obtained. The apparent ED_50_ (1) and ED_50_ (2) value for VPA is ~0.1 mM and ~0.5 mM, respectively, as indicated by the two arrows, see Figure 5A. See M&M for the calculation of ED_50_ and IC_50_ values. Data are presented as mean ± SD from 3 independent experiments (10 replicates each). All doses of VPA and TSA used in this study reached significance (*p* ˂ 0.05, by ANOVA and post hoc Student Newman–Keuls test) from the corresponding value in control (LPS + IFNγ).

The results in Figure 5A demonstrate that acute treatment with various concentrations of VPA induced an overall concentration-related stimulation of IL-1β from maximally stimulated J774A.1 cells at 24 h. Acute treatment of J774A.1 cells with low doses (0.075 mM–0.15 mM) of VPA followed by subsequent maximal stimulation induced a significant increase (*p* < 0.05; Figure 5A) in levels of IL-1β (with an apparent ED_50_ value of ~0.1 mM, indicated by the arrow, see Figure 5A). It was observed that higher concentrations of VPA (0.6 mM) significantly potentiated (approximately 400% more) the levels of IL-1β from J774A.1 cells (with an apparent ED_50_ value of ~0.5 mM, indicated by the arrow, see Figure 5A). The different concentrations of VPA used in this study exhibit a very interesting kinetic profile of stimulatory activity. It was observed that the lower (0.15 mM–0.3 mM) and higher (0.6 mM–1.2 mM) concentrations responsible for the increase in the levels of IL-1β reached two saturation points on the overall concentration-response curve (Figure 5A). We hypothesize that the kinetic effect may be due to two different stimulatory effects. We noted this interesting kinetic observation with VPA, which warrants further investigation.

Acute treatment with various concentrations of TSA induced multiphasic concentration-dependent stimulatory effect on the levels of IL-1β from maximally stimulated J774A.1 cells at 24 h. At the 12.5 nM–25 nM concentration range (see Figure 5B), acute treatment of J774A.1 cells with TSA with subsequent maximal stimulation reached an apparent saturation point. Similar to the effect of VPA on IL-1β, 50 nM and 200 nM of TSA significantly potentiated (approximately 400% more) the levels of IL-1β from J774A.1 cells. Despite the apparent multiphasic nature of this concentration-response curve in terms of stimulating the release of IL-1β, overall, all concentrations of TSA with subsequent maximal stimulation induce a significant stimulatory effect (*p* < 0.05; Figure 5B) from J774A.1 macrophages. Because the multiphasic dose-response curve appears to be much more complex in terms of the underlying mechanism, we consider it inappropriate to derive any ED_50_ for this dose-response curve.

## 3. Discussion

Contrary to our hypothesis, we found that acute treatment with VPA followed by maximal stimulation produced dose-dependent increases in levels of all three cytokines, TNFα (Figure 2A and Figure 3A), NO (Figure 4A) and IL-1β (Figure 5A), over the time course used in our studies. On the other hand, acute pretreatment with TSA showed mixed results. Similar to VPA, TSA also induced dose-dependent increases in the levels of IL-1β (Figure 5B) from maximally stimulated macrophages. However, TSA’s effect on TNFα (Figure 2B and Figure 3B) and NO (Figure 4B) levels exhibited a biphasic response. At low concentrations, TSA increased the levels of both TNFα and NO but had the opposite effect at high concentrations. This study sheds new light on the acute effect of VPA on cytokine levels in macrophages that may partially explain (i) the reduction in the number of resident macrophages in the murine VPA model of autism and (ii) the apparently occasionally conflicting data in the literature that may partly be explained by acute or short-term exposure versus chronic or long-term exposure to HDACi. In particular, the findings of this study suggest that using HDACis as anti-inflammatory agents may initially enhance the inflammatory state depending on the class, dose and time of exposure to the HDACi. 

As alluded to in the Introduction, NF-ĸB is a central player in the immune response because it regulates inflammatory genes, including iNOS, IL-1β and TNFα. For example, in murine N9 microglia, TSA clearly potentiated the LPS-induced proinflammatory cytokines TNFα, IL-6 and NO by NF-ĸB when cells were simultaneously treated with TSA and LPS [29]. It was suggested that the potentiation of the inflammatory response might be regulated at the transcriptional level. p65, which forms one of the components of NF-ĸB, is potentiated when treated simultaneously with pan HDACi (TSA or SAHA) and LPS in murine RAW264.7 cells [29]. This correlated with increased proinflammatory cytokines, suggesting a role for NF-ĸBp65 in the inflammatory response. Although we have not elucidated the mechanism for potentiation of the inflammatory response in our studies, the increase in TNFα and NO production we observed in maximally stimulated macrophages in response to VPA is likely mediated by NF-ĸB.

TNFα is induced by various inflammatory stimuli and plays a critical role in inflammatory disease and immunological responses [30]. It is also a commonly used marker of inflammation and infection along with iNOS induction and IL-1β. Our findings of increased TNFα and NO by VPA (See Figure 2A, Figure 3A and Figure 4A) and the biphasic response to TSA in relation to TNFα levels (See Figure 2B, Figure 3B and Figure 4B) in maximally stimulated macrophages bear some similarities and important differences in the response to VPA and TSA in cell culture and several animal models of inflammation. For example, reduced levels of TNFα and NO have been reported when cells were exposed to VPA (pretreatment with 0.4 mM and 0.6 mM VPA for 48 h prior to stimulation with LPS for another 24 h) in rat mesencephalic neuron glial culture [7]. Similarly, in a rat spinal cord injury model, different doses of VPA, 150 mg/kg and 300 mg/kg, reduced both the transcript and protein levels of TNFα, IL-1β and iNOS in the spinal cord [31]. Likewise, in the preventative and therapeutic experimental autoimmune encephalomyelitis model (EAE), high doses of VPA (500 mg/kg) reduced transcript levels of TNFα, IL-1β and iNOS in the EAE spinal cord [4]. All those findings indicate that valproic acid has anti-inflammatory effects on TNFα, IL-1β and iNOS. Although our findings vary from those in the literature, the results may be due to acute exposure (1 h treatment) of macrophages to VPA. Time-wise, it appears that acute exposure might be insufficient for VPA to exhibit its anti-inflammatory effect. However, our results are consistent with those by Surronen et al. described below, showing similar effects with low doses of TSA. 

Multiple studies in the literature have reported mixed results with TSA depending on the treatment protocol and dose. Han et al. noted a reduction in TNFα at both the transcript and protein levels when murine bone marrow-derived macrophages (BMDMs) were pretreated with TSA for 1 h (acute) prior to stimulation with the stimulant LPS alone [32]. These results bear some similarities with our findings in that pretreatment with TSA prior to stimulation with LPS + IFNγ led to an overall dose-dependent reduction of TNFα (See Figure 2B and Figure 3B) over the time course of our study, indicating that TSA exhibits a general anti-inflammatory effect irrespective of the treatment protocol.

With regards to NO, in our studies, TSA-induced decrease in NO production from maximally stimulated J774A.1 cells occurred only at the highest concentration (100 nM and 200 nM; see Figure 4B). Our observations are consistent with studies from Yu et al., who showed a single highest dose (200 nM) of TSA reduced nitrite production from LPS + IFNγ stimulated RAW264.7 cells (50% reduction) and murine mesangial cells (72% reduction) [33]. This was attributed partly to hyperacetylation and its effect on NF-ĸB. Although similar stimulus conditions and a macrophage cell line were used in the study by Yu et al., the limitation of their study was that, unlike our studies where we carried out an extensive dose response of TSA, this study used only a single high dose. The limitation of our study was that we did not investigate the cytotoxic effects of TSA. However, Han et al. demonstrated that acute treatment of murine primary BMDM with TSA followed by subsequent stimulation with LPS for 24 h impacted cell viability at high concentrations (100 nM and 200 nM) [32]. Therefore, we cannot rule out the possibility that the anti-inflammatory effects of TSA at the 100 nM–200 nM concentration range in our studies and 200 nM in studies carried out by Yu et al. may be due to cytotoxic effects of TSA.

Acute treatment of murine immune cell line RAW264.7 and BMDM cells with different concentrations of TSA followed by subsequent stimulation with LPS alone for 24 h resulted in a dose-dependent reduction in nitrite production [32]. This is consistent with other existing literature that showed siRNA inhibition of HADC4 followed by stimulation with LPS reduced the mRNA levels of IL-1β, TNFα and iNOS in RAW264.7 cells [34]. On the other hand, in the same study, when HDAC2 was inhibited by siRNA, the gene expression of iNOS was upregulated upon LPS stimulation. However, protein levels were reduced, indicating the role of HADC4 and HDAC2 in the anti-inflammatory effect [34]. Their observation contrasts with our results, where we show an increase in nitrite production at all doses except 100 nM (Figure 4B). The difference in the two studies may likely be due to maximal stimulation with LPS + IFNγ since all other conditions were the same and IFNγ is known to induce NO production in murine macrophages [35]. Our findings that TSA pretreatment increases NO production from LPS + IFNγ stimulated macrophages at low doses (See Figure 4B) is consistent with the results of studies in microglial cells. Suuronen et al. reported increased NO production in microglial cell culture, primary microglial culture and glial neuron coculture when cells were stimulated simultaneously with TSA (15–25 nM) and LPS for 24 h [29]. This increase in NO production appears to involve NF-ĸB at the transcriptional level because NF-ĸB inhibitors CAPE and helenalin strongly inhibited the TSA-induced potentiation [29]. In the same study, more prolonged pretreatment with TSA showed anti-inflammatory effects similar to other HDAC inhibitors such as SAHA [29]. Our results demonstrate that increased TNFα and NO production may likely be due to maximal stimulation of macrophages and acute treatment with TSA and VPA is insufficient to provide anti-inflammatory effects. 

The effects of HDACi on IL-1β release have been largely overlooked in the current literature. In addition to TNFα, the proinflammatory cytokine IL-1β is also produced and secreted from stimulated macrophages. Our finding that acute exposure to both VPA and TSA (See Figure 5A,B) followed by maximal stimulation of macrophages potentiated IL-1β was quite surprising, particularly with TSA, as it is well known to be a more potent anti-inflammatory agent compared to VPA. Our findings are in contrast to the results of studies in BMDM cells derived from 6- to 10-week-old C57/BL6 mice. Han et al. reported a decrease in IL-1β when BMDM cells were pretreated with 25 nM TSA for 1 h, followed by stimulation with LPS alone [32]. Similarly, in a lethal post-influenza pneumococcal infection mouse model, TSA (1 mg/kg administered 1 h after pneumococcal infection and continued daily throughout the course of the experiment) significantly decreased IL-1β in serum 36 h after pneumococcal infection [36]. The limitation of their study is that it does not provide data on the effect of TSA on IL-1β at an early time point. Overall, these studies suggest that TSA can also reduce IL-1β levels depending on the treatment protocol and kind of stimulus. A similar reduction in IL-1β is also observed with VPA in animal models of injury and autoimmune diseases [4,31]. There is more limited literature on the effect of acute treatment of VPA on IL-1β production from stimulated macrophages in cell culture. Interestingly, our findings show that acute exposure to VPA and TSA potentiates IL-1β (Figure 5A,B) from maximally stimulated macrophages, indicating that pan HDACi appear to have diverse effects on different cytokines.

IL-1β is tightly regulated at multiple levels, including transcription, translation, cleavage and release of the active cytokine. Monocytes, upon stimulation with LPS, secrete only about 20% of IL-1β from secretory lysosomes slowly over a 24 to 48 h period, unless a powerful stimulus-triggered secretion takes place, which may be attributed to danger signaling molecules like ATP and HMGB1 [37,38,39]. Generally, in vivo, dying cells or cells like monocytes and platelets actively secrete ATP that accumulates at the site of inflammation, thus potentiating IL-1β secretion [39]. HDAC inhibitors like SAHA or ITF2357 have been shown to inhibit the exocytosis of IL-1β containing secretory lysosomes by hyperacetylating tubulin, thus disrupting microtubules and impairing lysosome exocytosis in human monocyte from healthy donors [40]. Tubulin undergoes deacetylation by either HDAC6 or type III HDAC SIRT2, implicating the role of HDACs in IL-1β secretion [41,42,43]. It should be noted that this anti-inflammatory effect of SAHA or ITF2357 was observed when cells were pretreated with HDACi for at least 4 h prior to stimulation with LPS. More recently, Chi et al. also elucidated the role of HDAC3 on IL-1β levels. HDAC3 translocates to the mitochondria and deacetylates mitochondrial trifunctional enzyme subunit α HADHA, leading to increased IL-1β production in murine peritoneal macrophages [44]. However, the exact mechanism by which HADHA regulates IL-1β levels is unclear. Although the mechanism underlying the potentiation of IL-1β has not been fully elucidated, evidence is accumulating that several mechanisms could account for the potentiation of IL-1β. (i) One possibility is that HDACis induce some yet uncharacterized protein which may lead to the release of ATP that may act in an autocrine manner to potentiate IL-1β. (ii) HDAC3 seems to be involved in the inflammatory pathway of several proinflammatory genes and this interesting pathway merits further elucidation. (iii) The kinetics of the inhibition of IL-1β secretion by HDACi reveals that at least 4 h of exposure to the drugs is required to achieve an inhibitory effect. No inhibition was observed when treatment with HDACi SAHA and ITF2357 was restricted to one hour prior to the addition of stimulus in human monocyte culture [40]. Our observations are consistent with their studies, except that we used VPA and TSA. Both VPA and TSA are pan HDACis like SAHA and it is likely that one-hour pretreatment is not sufficient to hyperacetylate tubulin, which may lead to the pro-inflammatory effect observed in our studies. 

Our studies have attempted to address some of the knowledge gap and resolve some of the apparent conflicting data in the literature on the pro- and anti-inflammatory effects of HDACi. Unlike the limited number of doses of HDACi used in previous studies our studies utilized an extensive dose response of the HDACis TSA and VPA combined with maximal stimulation, which is a realistic pathophysiological inflammation-inducing paradigm. A picture is emerging that response of macrophages to the pan HDACis VPA and TSA may partly be due to the chemical class of HDACi, dose, treatment paradigm and time of exposure. 

One important aim of this study was to determine the effect of acute treatment of various concentrations of VPA followed by subsequent maximal stimulation on cytokine levels to mimic the VPA murine model of autism. Contrary to our hypothesis and the existing literature, surprisingly, our results reportedly showed apparent increases in levels of TNFα (Figure 2A and Figure 3A), NO (Figure 4A) and IL-1β (Figure 5A) at all doses used in this study. Embryonic and early postnatal brain development is a carefully regulated process involving both neuronal and immune cells and a host of other molecules such as cytokines like gp130 and JAK-STAT proteins that have been better characterized than the rest [45]. Immune dysfunction is associated with both clinical and animal models of autism [46]. For example, the cerebral cortex of adult rats exposed to VPA during embryonic development showed significantly high mRNA expression of IL-1β, IL-6 and TNFα relative to those in the saline control and even though that study did not report on protein levels of these proinflammatory cytokines, it appears that VPA induces a state of inflammation long after the effect of the drug is diminished [47]. Previously we have shown that VPA reduced the resident macrophage population, namely microglia in the primary motor cortex in male mice, 13–17 days after our single injection of VPA (Embryonic day 13.5, 600 mg/kg VPA, acute treatment), particularly because VPA has a half-life of less than 1 h in mice [20,48]. This observation prompted us to investigate if the reduction in microglia may be due to cell death, a direct primary effect of VPA because HDACis at high concentrations are known to induce cell death. Accordingly, our unpublished results clearly show that VPA exposure during embryogenesis induces cell death to a more significant extent in neuronal precursor cells (NPCs) 24–96 h after injection. Very little cell death is observed in microglia (unpublished data) during this time course, indicating that microglia cell death may be occurring during postnatal development and may potentially be a secondary effect of VPA, by potentiallyincreasing cytokine levels. The latter observation of ours prompted us to use a cell culture model to determine the acute effect of VPA on cytokine levels from stimulated macrophages. Indeed, our results clearly demonstrate that VPA potentiates the release of high levels of proinflammatory cytokines TNFα (Figure 2A and Figure 3A), NO (Figure 4A) and IL-1β (Figure 5A) from maximally stimulated macrophages. Based on the data obtained in this study, we hypothesize that increased cytokine levels may be attributed to the combined effect of immunomodulatory properties of VPA and cell death in NPCs in our model. Cell death may potentially lead to uncontrolled release of danger-associated molecular patterns (DAMPS) from dying cells, which may further enhance the inflammatory responses from microglia, eventually leading to reduced microglial number in our VPA model of autism. Nevertheless, cell death may be both a consequence and cause of inflammation and is very difficult to distinguish, as seen in rheumatic disease and other inflammatory conditions [49].

The studies conducted in this paper have several limitations in addition to those already mentioned. Firstly, we did not determine the cell viability in the VPA and TSA-treated macrophages because it is important for us to understand the reduced microglial number in the VPA model of autism. Secondly, we carried out acute treatment with HDACi for only 1 h. Future experiments should carry out a systematic kinetic study pretreating cells with HDACi at different times to obtain a clearer picture distinguishing the acute and chronic effect of HDACi on inflammatory cytokines and cell viability. The third limitation is that we did not elucidate the mechanism of HDACis in acute treatment to investigate how they potentiate the inflammatory response. Future studies should investigate the mechanism of the proinflammatory effect of acute exposure to HDACi and the anti-inflammatory effect of chronic exposure to HDACi. 

## 4. Materials and Methods

### 4.1. Reagents

Sodium valproate (VPA) was purchased from MP Biomedical, LLC (Illkirch, France). Trichostatin A (TSA) was purchased from Sigma Aldrich, Burlington, MA, USA. Lipopolysaccharide (LPS), Escherichia coli serotype 0111: B4 and recombinant mouse gamma-Interferon (IFNγ) were obtained from Calbiochem, San Diego, CA, USA. The medium used for cell cultures was purchased from Gibco, Carlsbad, CA, USA. The cell line J774A.1 was purchased from the American Type Culture Collection (ATCC) Manassas, VA, USA. Mouse TNF-alpha (TNFα) and mouse IL-1β eta (IL-1β) ELISAs were obtained from BD Biosciences, San Diego, CA, USA. Except where noted, all other chemicals were obtained from Sigma Aldrich at the highest grade possible. 

### 4.2. Cell Culture and Treatments

J774A.1 cells were cultured in DMEM media containing 4.5 g/L glucose and L-Glutamine supplemented with 10% heat-stimulated fetal bovine serum (FBS), 100 units/mL penicillin and 100 µg/mL streptomycin and maintained in 5% CO_2_ at 37 °C. For treatment, cells (from passage numbers 5 to 15) were plated in 96-well plates at a density of 5 × 10^4^ cells/well in media and allowed to adhere for 24 h. The cells were then treated with VPA, TSA or vehicle prior to activation with 1 µg/mL LPS and 1 ng/mL IFNγ as described in the figure legends. Concentrations of VPA and TSA used for these experiments were consistent with other published reports [7,50,51]. The dose-response used in this study ranged from 0.075 to 1.2 mM for VPA and 6.5 nM to 200 nM for TSA. The concentration used for VPA treatment is consistent with the therapeutic range used for human therapy (40–100 µg/mL or 0.28–0.69 mM VPA [52].

### 4.3. TNF-Alpha and IL-1βeta ELISAs

Mouse TNFα and IL-1β in cell culture supernatants were measured by ELISA (BD bioscience) as described by the manufacturer. All absorbance measurements were made using the SPECTRA max 340 PC Molecular Devices microplate reader at 450 nm.

### 4.4. Nitrite Assay

Nitrite concentrations were measured as an indirect measurement of nitric oxide (NO) using a modified method of the Griess assay [53]. 

### 4.5. ED_50_ and IC_50_ Calculations

The apparent ED_50_ value for VPA (see Figure 2A and Figure 5A) was determined by calculating the 50% difference between the highest and lowest percentage of the respective cytokine released by the stimulated macrophages resulting from the acute treatment of VPA and dropping a perpendicular line from this point on the plot to the X-axis and taking the antilog of the intercept value on the X-axis. The apparent IC_50_ value for TSA (see Figure 2B, Figure 3B and Figure 4B) was determined by calculating the percentage needed to inhibit response by 50% from the highest percentage t of the respective cytokine released by the stimulated macrophages resulting from the acute treatment of TSA and dropping a perpendicular line from this point on the plot to the X-axis and taking the antilog of the intercept value on the X-axis. 

### 4.6. Statistical Analysis 

Statistical analysis was conducted using InStat 3 (Graphpad). To compare various treatments, we used one-way ANOVA with a Student Newman–Keuls post hoc test. When comparing two specific groups, Student’s *t*-test was used. For data points in each graph where the diameter of the symbol is larger than the magnitude of the error bar, the error bars could not be shown.

## 5. Conclusions

While our observation that acute treatment with VPA shows that increased levels of cytokines may have some implication for the mechanism of reduction of microglia in our murine VPA model of autism, work is underway in our lab to further understand the mechanism of VPA in this model. In addition, our observation that acute exposure to extensive doses of HDACi induces apparent contradictory inflammatory responses depending on the class, dose and treatment protocol may have a pathophysiological implication; the molecular mechanisms underlying these effects remain to be fully elucidated. Future experiments should investigate a whole gamut of inflammatory molecules under different treatment protocols to understand the effect of HDACi on immune cells. Detailed studies to define specific HDAC upregulation, histone and nonhistone protein involvements under different conditions are now underway in our lab. Nevertheless, what has been emerging is that at least some of the mechanisms underlying immune regulation implicate both histone and nonhistone proteins. These epigenetic and downstream signaling mechanisms are important and relevant to many clinical states in which inflammation persists and, as such, merit further investigation. 

## Data Availability

Not applicable.
